# Case Report: Laparoscopic repair of a recurrent, huge, inguinoscrotal vesicoureteral hernia: a case report and review of the literature

**DOI:** 10.3389/fsurg.2025.1664253

**Published:** 2025-11-11

**Authors:** Shunhua Tian, Guilin Min, Fan Yang, Qingfeng Tan, Hongliu Chen

**Affiliations:** Department of Gastrointestinal Surgery, Minda Hospital of Hubei Minzu University, Enshi, Hubei, China

**Keywords:** vesicoureteral hernia, laparoscopic, CT urography, case report, recurrent hernia

## Abstract

Inguinal hernia repair is a routine operation. However, it is rare for the organs of the urinary system to prolapse as the contents of the hernia. An 80-year-old obese male with a history of three prior open inguinal hernia repairs presented with a recurrent large scrotal mass and severe right hydronephrosis. Preoperative Computed Tomography urography (CTU) revealed a giant inguinal vesicoureteral hernia involving the bladder and ureter. The patient underwent transabdominal preperitoneal repair (TAPP) with intraoperative identification of herniated bladder-ureter components facilitated by bladder catheter saline infusion and ureteroscopic assistance. Postoperative CTU at 1 week demonstrated complete reduction of herniated viscera and resolution of hydronephrosis. No complications occurred, and 6-month follow-up confirmed sustained recovery without recurrence. This case provides valuable insight into preoperative diagnostic difficulties and the intra- and postoperative management of an inguinal vesicoureteral hernia in a multiple relapsed old man, highlighting the importance of accurate diagnosis and appropriate surgical intervention in the treatment of this disease.

## Introduction

Less than 7% of patients with inguinal bladder hernia (IBH) are diagnosed preoperatively (1), while the ureteral hernia diagnosis rate is about 1% (2). Up to 12% of patients with IBH experience intraoperative bladder injury if not diagnosed preoperatively (3). If a patient also has a combined ureteral hernia, a intraoperative repair is very difficult. We report a case of an 80-year-old male patient who underwent three open repair operations and presented with a large scrotal mass with severe right hydronephrosis. Preoperative computed tomography urography (CTU) examination demonstrated a large inguinal scrotal vesicoureteral hernia. We performed laparoscopic transperitoneal extraperitoneal hernia repair (TAPP) with good results. This work was reported in line with the SCARE 2020 criteria (4).

## Case presentation

An 80-year-old obese man of Chinese ethnicity presented himself to our hospital with a painless mass in the right groin and scrotal area for the past 6 months. He denied any history of fever, dysuria, or recent episodes of abdominal or back pain. His medical history was significant for benign prostatic hyperplasia and three previous open repairs for a right inguinal hernia, the first performed at the age of 50. The exact surgical techniques of the prior repairs were unknown. The most recent herniorrhaphy was performed over one year prior to this admission. The patient reported no chronic pain following the previous surgeries and stated that the hernia had always been reducible, with no history of incarceration or strangulation prior to the onset of the current, persistent mass. He had no known drug allergies and was a non-smoker. His home medications included tamsulosin for his prostatic hyperplasia.

**Figure 1:**
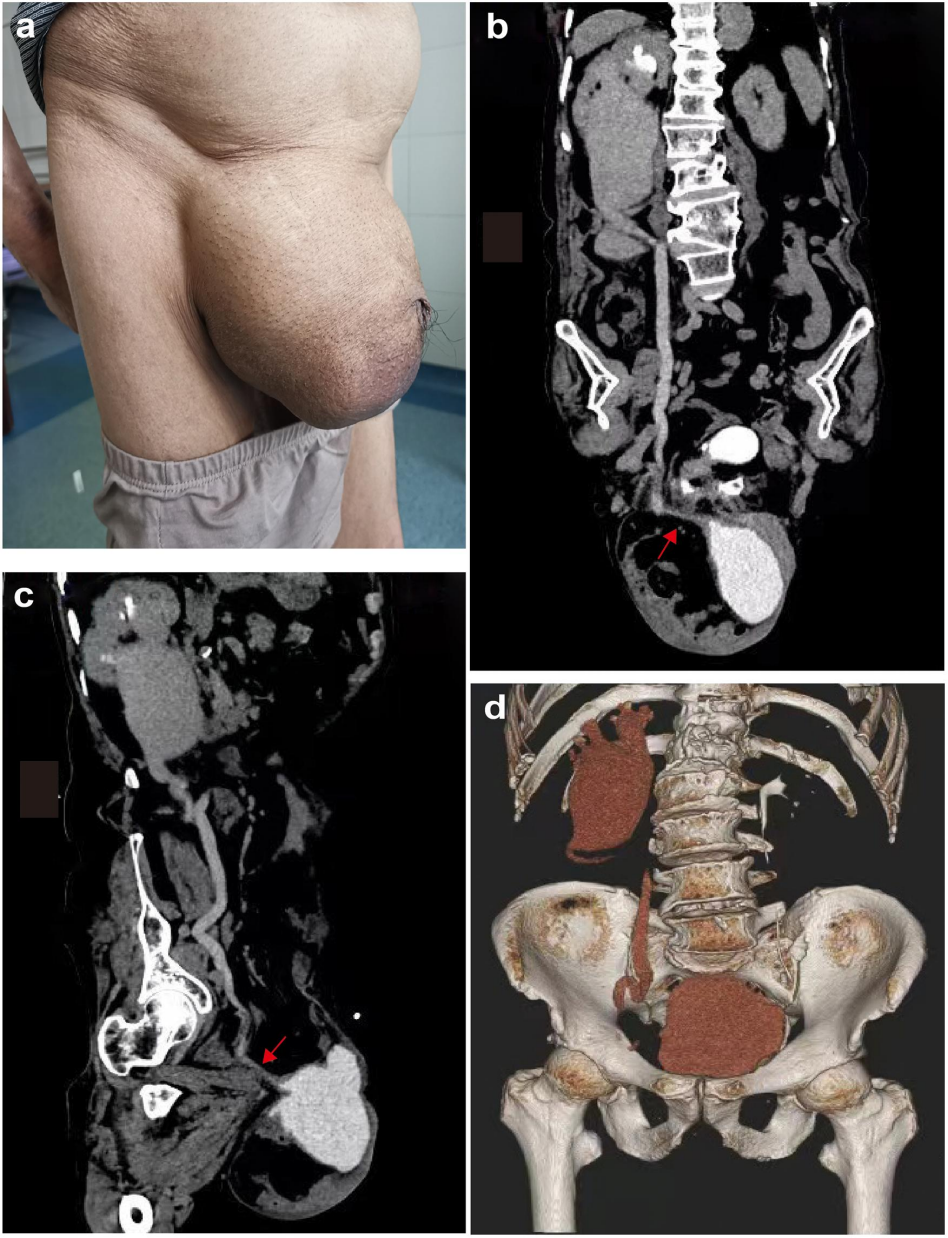
**(a)** Preoperative appearance of the right inguinal region **(b,c)** preoperative and postoperative CT urography (CTU) of the patient. CTU demonstrates a right inguinal hernia, intestinal and omentum hernia, bladder, and distal ureteral hernia, dilated right ureter, and severe right hydronephrosis. The red arrow marks the herniated right distal ureter. **(d)** The CTU was repeated 7 days after surgery, and the bladder and right ureter were repositioned.

On physical examination, his vital signs were stable and afebrile. Calculation of his body mass index (BMI) revealed obesity (30 kg/m^2^). Abdominal examination revealed a surgical scar in the right groin area, with about 20 × 15 cm^2^ large masses in the right groin and scrotum, which could be reduced by supine or manual reduction, as shown in Figure 1a. Based on the European Hernia Society (EHS) classification for inguinoscrotal hernias, the case was categorized as S1. It was further defined as Type 3 according to the Campanelli classification system

Preoperative and postoperative laboratory results are shown in Table 1. Preoperative color ultrasound showed a right inguinal hernia, severe right hydronephrosis, and prostatic hyperplasia. CTU demonstrated right inguinal hernia, herniation of the intestine, omentum, most of the bladder, and distal ureter, right ureter dilation, and severe right hydronephrosis, as shown in Figures 1b,c. The CTU did not reveal any evidence of radiopaque mesh material from previous repairs. Following the CTU findings, a targeted history was retaken. The patient then described a two-stage pattern of urination: an initial normal stream, followed by a need to manually compress the scrotal mass to complete voiding, after which the mass would noticeably reduce in size.

**Table 1:** Comparison of main laboratory examination results before and after surgery.

Laboratory inspection	Preoperative	After 1 week	Reference ranges
HGB (g/L)	112	106	130–175
WBC (1** ×** 10^9^/L)	9.29	8.27	3.50–9.50
FR-CRP (mg/L)	106.60	53.41	0–6.00
BUN (mmol/L)	5.75	3.33	1.70–8.30
Cr (μmol/L)	80	78	40–110
CysC (mg/L)	1.76	1.17	0.54–1.25
Urinalysis	Negative	Negative	Negative

HGB, hemoglobin; WBC, white blood cell count; CRP, C-reactive protein; FR-CRP, full range C-reactive protein; BUN, blood urea nitrogen; Cr, creatinine; CysC, cystatin C protein.

Based on the preoperative workup and history of multiple open surgeries, the laparoscopic (TAPP) approach was recommended for this complex case. The procedure was performed under general anesthesia at our regional teaching hospital by a senior consultant surgeon with over a decade of experience in advanced laparoscopic surgery. Upon intraoperative exploration, the preperitoneal plane was significantly scarred and fibrotic, consistent with multiple previous surgeries. However, no remnants of prior synthetic mesh were identified. We entered the preperitoneal space and first looked for the position of the ureter, especially the segment of the ureter that had moved down beyond the hernia sac, to identify its relationship to the hernia sac and avoid injury while freeing the peritoneal flap and performing a transection of the hernia sac. Secondly, an additional 5-mm puncture hole was added to the regular TAPP puncture hole to facilitate retraction exposure. After the reduction of the bladder and ureter, we tried performing a ureteroscopy and expected to insert a double J tube. However, the right ureter could not be entered due to its distortion, and catheterization failed. Once the right ureteral opening was confirmed to be free of abnormality, we left an 11 × 16-cm anatomically contoured polypropylene mesh (BARD® 3DMAX^TM^ mesh, Davol, Cranston, RI, USA) in place to complete the abdominal wall reinforcing. The mesh was secured using interrupted non-absorbable sutures (Ethibond Excel® Polyester Suture, Ethicon, US). Key fixation points included: Medially to the stable stump of the Cooper's ligament. Superolaterally to the firm scar tissue and the anterior abdominal wall musculofascial structures.

The patient's postoperative recovery was uneventful. He was started on a liquid diet on postoperative day 1, which was advanced to a regular diet as tolerated. Analgesia was managed effectively with oral non-opioid medications. He remained afebrile, and his surgical wounds healed well without signs of infection. The patient was ambulatory early and discharged on postoperative day 7 after confirming his ability to void spontaneously and manage pain with oral analgesics. Discharge instructions included advice on avoiding heavy lifting for 6 months and arrangements for follow-up urologic evaluation to monitor the resolution of hydronephrosis.

A follow-up CTU one week after surgery demonstrated significant reduction in the herniation of the bladder and ureter, with concomitant improvement in ureteral dilation and right renal hydronephrosis (Figure 1d). The patient completed the established treatment plan and was discharged. At the six-month clinical and ultrasonographic follow-up, the patient reported no discomfort, recurrence of the hernia, or urinary symptoms. Repeat ultrasound confirmed the complete resolution of the right hydronephrosis.

## Discussion

The 2018 International Inguinal Hernia Guidelines (5) state that nearly one-third of male patients develop an inguinal hernia during their lifetime, yet inguinal bladder hernias account for only 1%–4% (1), and concurrent ureteral hernias are even rarer. Since the first case of inguinal ureteral hernia was reported by Leroux et al. in 1880, more than 140 cases of inguinal ureteral hernia have been reported to date (6–9). Due to the low incidence, preoperative identification remains challenging, with fewer than 7% of IBH cases diagnosed prior to surgery (1). Moreover, the preoperative diagnosis of ureteral hernia is even lower at about 1% (2). One study reported that merely 1 out of 139 individuals with inguinoscrotal hernia received preoperative ultrasonographic assessment for ureteral dilation (10). The typical two-stage voiding symptoms were not mentioned to the clinician in our case. The fact that the patient did exhibit this clinical symptom when asked again after the CTU verified that the diagnosis might have been attributed to the clinician's lack of awareness of such rare events. The presence of ureteral obstruction indicators, including hydronephrosis or ureteral dilation, in patients with inguinal hernias should raise clinical suspicion for possible ureteral herniation. Many patients with a preoperative diagnosis of vesicoureteral hernia are often incidentally diagnosed by CT, magnetic resonance imaging, positron emission tomography-computed tomography, single-photon emission computed tomography/computed cosmography, and other imaging studies due to various comorbidities, such as ureteral stones, bladder stones, hydronephrosis, acute renal dysfunction, intestinal obstruction, and tumors (11–14).

Multiple risk factors have been associated with vesicoureteral herniation, including male sex, obesity, age over 50 years, previous inguinal hernia repair, pelvic injury, particularly to the ligaments supporting the bladder, bladder obstruction (such as prostatic hypertrophy), urethral stricture, prostatitis, bladder neck stricture, chronic untreated encopresis, pathological changes in the bladder wall (such as hypomyelination), bladder diverticulum, chronic inflammation, and malignancy (15–18). Our patient, with a BMI of 30 and three ipsilateral inguinal hernia repairs since the age of 50 years, exhibited the characteristic two-stage voiding pattern: initial emptying of the orthotopic bladder followed by manual compression of the scrotal mass to evacuate urine from the herniated bladder component. Additionally, complications of inguinal scrotal vesicoureteral hernias include vesicoureteral reflux, sepsis, bladder stones, unilateral and/or bilateral hydronephrosis, renal failure, and strangulation with ischemia and infarction of the bladder (19). Detailed preoperative imaging of all patients with inguinal hernias is not possible, but if a patient has the aforementioned high-risk factors and develops these complications, it warrants a high degree of vigilance with further preoperative imaging evaluation. It has been suggested that cystourethrography is the gold standard for the diagnosis of IBH (17, 18, 20). We believe that CTU is a good method for diagnosing vesicoureteral hernias, especially in patients with massive scrotal bladder hernias where the distal ureter may subsequently migrate downward. Furthermore, it is more advantageous to know the condition of the distal ureter.

Inguinal ureteral hernias are anatomically classified into two types: paraperitoneal and extraperitoneal (21). The paraperitoneal type is more common, accounting for approximately 80% of all cases (9). In paraperitoneal hernia, the bladder and bowel form the visceral component of the hernia sac. However, there is no hernia sac in an extraperitoneal type, and the enlarged component of the scrotum contains retroperitoneal adipose tissue and the ureter (21). The extraperitoneal form is thought to be associated with abnormal development of the urinary system, such as adhesion of the gubernaculum testis to the ureter and wandering kidney (22). Because of their low incidence, vesicoureteral hernias are often underrecognized by surgeons. Additionally, there are no clear guidelines on how to manage vesicoureteral hernias safely and effectively.

Preoperative diagnosis of vesicoureteral hernias is essential to avoid bladder and ureteral injury. In the case of large scrotal hernias, transection of the hernia sac is often required. If vesicoureteral hernias are not diagnosed preoperatively, the non-filling bladder and ureter are easily injured during surgery and are not easily detected, making postoperative management very difficult.

The general surgical treatment of vesicoureteral hernias involves the retraction of the prolapsed bladder and ureter into the retroperitoneal cavity in the inguinal region. Otherwise, postoperative hydronephrosis may develop due to torsion and bending of the ureter, eventually leading to ureteral rupture. In this group of patients, open repair was previously reported, and laparoscopic repair has been increasingly reported in recent years, with occasional reports of robotic repair procedures (23). It is important to investigate how to successfully return the herniated bladder and ureter while avoiding injury during surgery in patients with inguinal vesicoureteral hernias. One report has shown that the ureter could be identified during surgery by careful observation of its peristaltic movements and by aspiration of its contents through puncture (2). We intraoperatively filled the bladder with saline, which facilitated the identification of anatomical structures. At the same time, the assistant could lift the saline-filled bladder upward from the base of the scrotum to facilitate the search for a gap and free bladder repositioning, which we think is particularly important.

## Conclusion

This case provides valuable insight into preoperative diagnostic difficulties and intra- and postoperative management of an inguinal vesicoureteral hernia in an elderly male patient with multiple relapses, highlighting the importance of accurate diagnosis and appropriate surgical intervention for this disease. TAPP repair of a vesicoureteral hernia is safer and minimally invasive, especially in the case of multiple relapses, although it requires some surgical techniques different from conventional TAPP repair.

## Data Availability

The original contributions presented in the study are included in the article/Supplementary Material, further inquiries can be directed to the corresponding authors.
